# Identification of acetylcholinesterase inhibitors from traditional medicinal plants for Alzheimer's disease using *in silico* and machine learning approaches†

**DOI:** 10.1039/d4ra05073h

**Published:** 2024-10-31

**Authors:** Md. Tarikul Islam, Md. Aktaruzzaman, Ahmed Saif, Al Riyad Hasan, Md. Mehedi Hasan Sourov, Bratati Sikdar, Saira Rehman, Afrida Tabassum, Syed Abeed-Ul-Haque, Mehedi Hasan Sakib, Md. Muntasir Alam Muhib, Md. Ali Ahasan Setu, Faria Tasnim, Rifat Rayhan, Mohamed M. Abdel-Daim, Md. Obayed Raihan

**Affiliations:** a Department of Genetic Engineering and Biotechnology, Faculty of Biological Science and Technology, Jashore University of Science and Technology Jashore 7408 Bangladesh; b Laboratory of Advanced Computational Neuroscience, Biological Research on the Brain (BRB) Jashore 7408 Bangladesh; c Department of Pharmacy, Faculty of Biological Science and Technology, Jashore University of Science and Technology Jashore 7408 Bangladesh aktaruzzaman.phar@gmail.com +88019295912; d Department of Pharmacy, Faculty of Science, University of Rajshahi Rajshahi 6205 Bangladesh; e Department of Microbiology, Faculty of Biological Science, University of Rajshahi Rajshahi 6205 Bangladesh; f Department of Pharmaceutical Sciences, College of Health Sciences and Pharmacy, Chicago State University Chicago IL USA; g Department of Biomedical Engineering, Jashore University of Science and Technology Jashore 7408 Bangladesh; h Department of Genetic Engineering and Biotechnology, Faculty of Life and Earth Sciences, Jagannath University Dhaka 1100 Bangladesh; i Department of Microbiology, Faculty of Biological Science and Technology, Jashore University of Science and Technology Jashore 7408 Bangladesh; j Department of Biological Sciences, Bose Institute Unified Academic Campus, EN-80, Salt Lake, Sector V, Bidhannagar Kolkata 700091 West Bengal India; k Faculty of Pharmaceutical Sciences, Lahore University of Biological and Applied Sciences Lahore Punjab Pakistan; l Department of Genetic Engineering and Biotechnology, University of Rajshahi Rajshahi 6205 Bangladesh; m Department of Pharmaceutical Sciences, Pharmacy Program, Batterjee Medical College P. O. Box 6231 Jeddah 21442 Saudi Arabia; n Pharmacology Department, Faculty of Veterinary Medicine, Suez Canal University Ismailia 41522 Egypt

## Abstract

Acetylcholinesterase (AChE) holds significance in Alzheimer's disease (AD), where cognitive impairment correlates with insufficient acetylcholine levels. AChE's role involves the breakdown of acetylcholine, moderating cholinergic neuron activity to prevent overstimulation and signal termination. Hence, inhibiting AChE emerges as a potential treatment avenue for AD. A library of 2500 compounds, derived from 25 traditionally used medicinal plants, was constructed using the IMPAAT database of traditional medicinal plants. The canonical SMILES of these compounds were collected and underwent virtual screening based on physicochemical properties, with subsequent determination of IC_50_ values for the screened compounds followed by analysis using machine learning (ML). Subsequently, a molecular docking study elucidated both binding affinity and interactions between these compounds and AChE. The top three compounds, exhibiting robust binding affinities, underwent MM-GBSA analysis for molecular docking validation, succeeded by pharmacokinetics and toxicity evaluations to gauge safety and efficacy. These three compounds underwent MD simulation studies to assess protein–ligand complex conformational stability. Additionally, Density Functional Theory (DFT) was employed to ascertain HOMO, LUMO, energy gap, and molecular electrostatic potential. Among 2500 compounds, physicochemical properties-based virtual screening identified 80 with good properties, of which 32 showed promising IC_50_ values. Molecular docking studies of these 32 compounds revealed various binding energies with AChE, with the best three compounds (CID 102267534, CID 15161648, CID 12441) selected for further analysis. MM-GBSA studies confirmed the promising binding energies of these three compounds, validating the molecular docking study. Further, the MD simulation studies have confirmed the structural and conformational stability of these three protein–ligand complexes. Finally, DFT calculations revealed favorable chemical features of these compounds. Thus, we can conclude that these three compounds (CID 102267534, CID 15161648, CID 12441) may inhibit the activity of AChE and can be useful as a treatment for Alzheimer's disease.

## Abstract 2

Acetylcholinesterase (AChE) holds significance in Alzheimer's disease (AD), where cognitive impairment correlates with insufficient acetylcholine levels.

## Graphical abstract

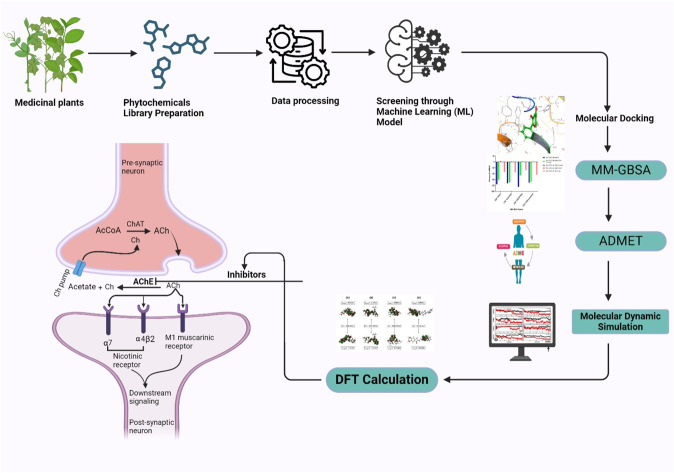

## 1. Introduction

Alzheimer's disease (AD) is a neurodegenerative disease that results in progressive declines in memory, thinking, and social abilities.^1^ Currently, there is no disease-modifying treatment available for this condition, which is one of the most challenging healthcare problems of our time. The discovery of disease-modifying treatment strategies for Alzheimer's disease remains a topic of ongoing research.^2^ As the global population continues to age, the prevalence of Alzheimer's disease (AD) is steadily increasing, highlighting the necessity to develop disease-modifying therapies capable of slowing down or halting the progression of the disease.^3^ The pathogenesis of AD is characterized by the involvement of a number of pathways and processes. One of these pathways is the acetylcholinesterase (AChE) pathway, which results in a progressive loss of neuronal communication.^4^ In Alzheimer's disease progression, the decline in acetylcholine levels due to the degeneration of cholinergic neurons contributes to cognitive impairment.^5^ Reduced acetylcholine availability disrupts synaptic transmission, exacerbating memory deficits and cognitive decline over time.^6^ As a result of reduced acetylcholine (ACh) activity in the hippocampus, memory deficits are believed to be caused by the degeneration of cholinergic neurons. The brain exhibits a severe dysregulation of the AChE pathway as a characteristic feature of AD. AChE is a catabolic enzyme that contributes to the breakdown of ACh in the brain, and is considered a disease-modifying therapeutic strategy in AD as well.^7,8^ Several drugs were developed and approved for treating AD symptoms, including tacrine, donepezil, rivastigmine, and galantamine. However, they have several side effects including syncope, nausea, vomiting, seizures, dizziness, and diarrhea.^9^ Medicinal plants produce an endless range of primary and secondary compounds as a result of secondary metabolism, which leads to greater chemical diversity than other natural sources with pharmacological activity.^10^ Researchers have shown a strong interest in investigating traditional medicinal plants, their constituents, and even their mixtures for the development of medications for treating diseases.^11^ The chemical constituents in them are utilized for the development of drugs because of their less harmless effects than synthesized chemical drugs.^12^ According to reports, phytochemical compounds such as alkaloids, flavonoids, lignans, tannins, triterpenes, polyphenols, and sterols, each exhibit diverse pharmacological activities including anticholinesterase, anti-inflammatory, anti-amyloidogenic, antioxidant, and hypolipidemic effects.^13,14^ Therefore, our primary focus is to identify natural anti-Alzheimer's bioactive inhibitors targeting the acetylcholinesterase (AChE) enzyme from twenty-five traditionally used medicinal plants using computational approaches.

## 2. Materials and methods

### 2.1. Phytochemical library preparation

Traditional medicinal plants have been used for the isolation of essential natural bioactive compounds that are an important source of both preventive and curative medical treatment.^15^ A library of 2500 compounds from 25 traditionally used medicinal plants was constructed using the IMPAAT (Indian Medicinal Plants, Phytochemistry, and Therapeutics) database. The local, and scientific names and traditional uses of these plants are presented in ESI Table 1.†

### 2.2. Filtering undesirable sub-substructures with physiochemical analysis

Physiochemical analysis provides a means for removing unwanted components from virtual screenings, based on their chemical features, to reduce false positive results and side effects.^16^ The canonical SMILES of these 2500 compounds were collected and subjected to virtual screening based on physicochemical properties. Phenol–sulfonamides, phenol–esters, rhodanines, curcumin, hydroxyphenylhydrazones, enones, catechols, toxoflavin, isothiazolones, analines, and quinones were among the most frequently detected undesirable organic compounds. Using RDKit v2023.03, different molecular, and physicochemical properties of the phytochemicals were calculated, including molecular weight, hydrogen bond acceptors and donors, rotatable bonds, *M* log *P*, TPSA, molar refractivity, heteroatoms, and aromatic rings. The physiochemical parameters were set as follows: molecular weight between 200 and 480, *M* log *P* from −0.4 to 4.15, heteroatoms > 1, molar refractivity between 40 and 130, and TPSA up to 131.6. These criteria are commonly used as filters to exclude chemicals in the initial stages of computer-aided drug discovery. Our library contains 2500 canonical smiles of compounds, of which 80 canonical smiles (1.77%) passed the physicochemical properties-based virtual screening according to the criteria, and all the data are presented in ESI File 1.†

### 2.3. Re-screening through machine learning (ML)

Previously screened compounds were then subjected to machine learning techniques for determining IC_50_ values. The canonical smiles of these compounds were selected for re-evaluation using a machine learning approach with the Light Gradient Boosting Machine (LightGBM) to anticipate models utilizing the CHEMBL220 dataset, which comprises acetylcholinesterase inhibitors (AChE).^17^ To represent the molecular structure of each compound, PubChem fingerprints were generated using the PaDEL software, resulting in a total of 881 molecular fingerprints per compound. The dataset was separated into a test set, and a training set using an 80/20 ratio to train and evaluate the model, respectively. To reduce noise and improve model interpretability, Recursive Feature Elimination (RFE) was applied for feature selection.^18^ The RFE method was employed with LightGBM as the base estimator. In this case, the number of selected features was set to 10, which was chosen after several experiments to optimize performance. To further enhance the model's performance, hyperparameter tuning was performed using Grid Search with 5-fold cross-validation. The hyperparameters optimized during this process included: “num_leaves”, “learning_rate”, “n_estimators”, “max_depth”, and “feature_fraction”.^17,18^ We evaluated the model's accuracy using multiple machine learning metrics such as *R*^2^ and MSE. To determine *R*^2^, MSE (mean square error), and pIC_50_, we used the following equations (eqn (1)–(3)).

1 *R*^2^ = 1 − Σ(*Y*_*i*_ − *Ŷ*_*i*_)^2^/Σ(*Y*_*i*_ − *Ȳ*)^2^

2 MSE = 1/*n*Σ(*Y*_*i*_ − *Ŷ*_*i*_)^2^

where *Y*_*i*_ indicates the actual values, *Ŷ*_*i*_ represents the predicted values, and *Ȳ* the mean of the actual values.

The predicted pIC_50_ score was determined using this equation:

3 pIC50 = log_10_IC_50_ (M) = 9 − log_10_IC_50_ (nM)

The model was subsequently applied to re-screen the 80 compounds, which were converted into PubChem fingerprints, as detailed in ESI File 2.†

### 2.4. Protein preparation

**Fig. 1:**
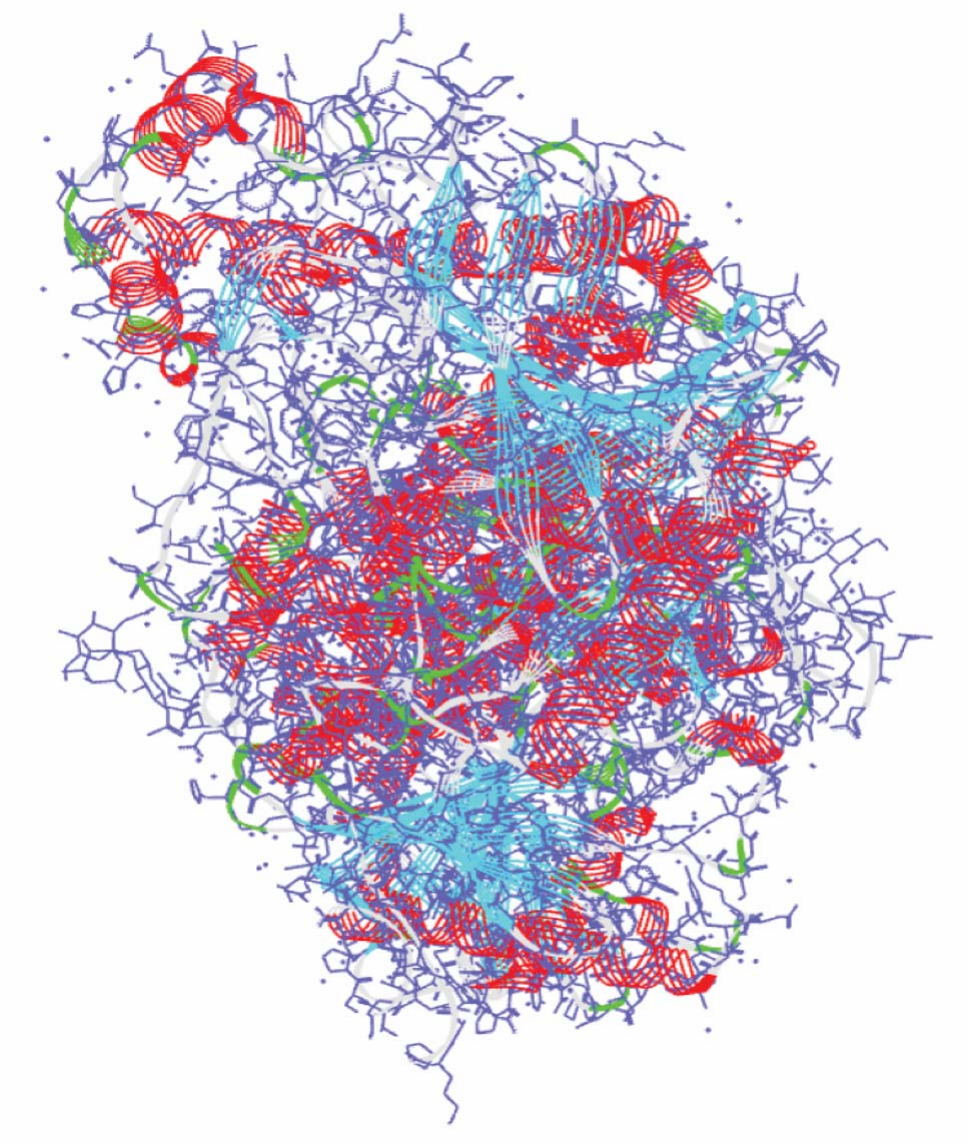
The 3D X-crystal structure of acetylcholinesterase enzyme (PDB ID: 4EY7).

The 3D structure of human acetylcholinesterase (PDB ID: 4EY7) was obtained from the Protein Data Bank (https://www.rcsb.org/) shown in Fig. 1. The amino acid sequence length of human acetylcholinesterase protein is 542 with resolutions of 2.35 Å. Using the protein preparation wizard of the Schrodinger Suite 2022-4, water, co-factors, unnecessary chains, and metals, were eliminated while hydrogen and missing site chains were added.^19^ The system was optimized by employing the OPLS3 force field.^20^

### 2.5. Ligand preparation

A total number of 32 re-screened phytochemicals from ML and these compounds along with the control drug (donepezil) were retrieved from the PubChem database (https://www.pubchem.mcbi.nlm.nih.gov/) in 2D and 3D SDF format. For preparing the ligand to conduct molecular docking, refinement, and processing were carried out using the Maestro v11.3 LigPrep module.^21,22^ Finally, the docking analysis was optimized using the OPLS3 force field.^20^

### 2.6. Active site prediction and receptor grid generation

Biological and biochemical active sites are the regions of an enzyme where substrate molecules bind and undergo chemical reactions. For this reason, determining the active site is a prerequisite before conducting molecular docking and subsequently grid box generation.^23^ By ensuring favorable catalytic microenvironments, the active site facilitates the formation of sufficient contact points between chemicals and the targeted enzymes, resulting in robust binding.^24^ This study used the FTSite server (https://ftsite.bu.edu/) to map the active site of the protein and generate a receptor grid to determine a phytochemical's binding affinity.^25^ To select the area for small molecules docking modeling, a grid box with dimensions of *X* = 13.99, *Y* = −44.1, and *Z* = 28.01 was generated for 4EY7.

### 2.7. Molecular docking study

Molecular docking is a mathematical method that determines the binding energies of small molecules to the target receptors.^26^ The binding energy of the target protein–ligand complexes was estimated based on molecular docking analysis. In this analysis, the Glide package v-8.8 and Maestro v-12.5.139 of the Schrödinger Suite were used to conduct molecular docking studies on a selection of phytochemicals. For docking, OPLS3 was used as a force field in standard precision (SP) mode to optimize the system.^20^

### 2.8. Post-docking MM-GBSA analysis

The molecular mechanics-generalized Born surface area (MM-GBSA) is calculated to determine the free energy of ligand binding for a combination between the phytochemicals and the protein using the Prime MM-GBSA program package.^27^ This analysis compares the relative binding free energies of the control compound and the identified ligands against the selected receptor.^28^ The negative MM-GBSA Δ*G* bind (NS), Δ*G* bind Coulomb (Coulomb energy), Δ*G* bind H-bond (hydrogen bond energy), Δ*G* bind lipo (lipophilicity energy), and Δ*G* bind vdW (van der Waals interaction energy) were considered.^29^

### 2.9. Pharmacokinetics and toxicity analysis

Pharmacokinetics and toxicity analysis are crucial in drug design for ensuring the safety, efficacy, appropriate dosing, and regulatory compliance of new drug candidates.^30^ Evaluation of the integrity and effectiveness of compounds through pharmacokinetic and toxicity properties should be done early in the drug design phase. To evaluate the pharmacokinetic features of the selected phytochemicals at an initial stage, we utilized the SwissADME server,^31^ an online tool available at (https://www.swissadme.ch/). This server provides an extensive analysis of the pharmacokinetics and characteristics of small molecules. Drug development and design require an assessment of toxicity. Therefore, we assessed the toxicity of our selected compounds using the ProTox-II server (https://tox.charite.de/protox_II).^32^

### 2.10. Molecular dynamic (MD) simulation study

MD simulation is an important and effective simulation technique to observe conformational changes where ligand–receptor complexes are facilitated to move over a specific time scale.^33^ A 250 ns period of MD simulation was conducted to observe the binding equilibrium state of protein–ligand complexes using the ‘Desmond v3.6 Program’ in Schrodinger (Academic version) on a Linux platform.^34^ A preset TIP3P water model was applied to operate the system, and an orthorhombic periodic boundary box shape with a diameter of 10 × 10 × 10 Å^3^ was adopted on both sides to maintain an appropriate volume and ions such as Na^+^ and Cl^−^ with a salt level of 0.15 M throughout the entire system for electrical neutralization. The whole system was stabilized using an OPLS3e force field.^35^ The temperature at 300.0 K and the pressure at 1.01325 bar were maintained throughout the simulation in the NPT (constant pressure–constant temperature) ensemble.

#### Simulation trajectory analysis

Schrodinger's Maestro interface version 9.5 was utilized to create each MD simulation snapshot. The simulation events were analyzed using the Simulation Interaction Diagrams (SID) of the Desmond module in the Schrodinger package. The stability and dynamic properties of the complexes were assessed by computing root mean square deviation (RMSD), root mean square fluctuation (RMSF), the radius of gyration (*R*_g_), solvent accessible surface area (SASA), protein–ligand contact (P–L contact), and principal component analysis (PCA).

#### RMSD analysis

RMSD analysis calculates the dislocation distance in a protein–ligand complex over a specific period.^36^ Protein-suited molecules of RMSD from all frames are calculated and compared to simulation times of 250 ns. The following formula (eqn (4)) should be used for calculating the RMSD.4
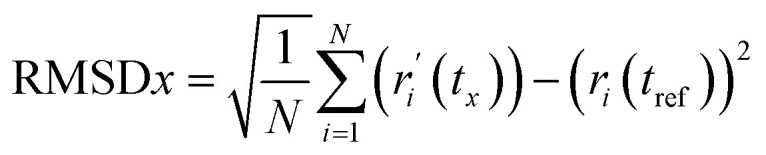
Here, *N* represents the number of selected atoms, *t*_ref_ denotes the reference or specified time, *r* indicates the specified atom's location in frame *x* after superimposition on the reference frame, and *t*_*x*_ indicates the duration of the recording intervals.

#### RMSF analysis

RMSF represents the local conformational changes within the protein structure.^37^ A protein's RMSF value can be determined using the following equation (eqn (5)) by multiplying its number of residues by its RMSF value.5
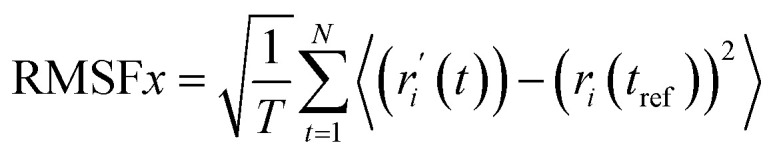
Here, *T* primarily refers to the trajectory time, *t*_ref_ is the reference or given time, *r* indicates the selected atoms' positions in frame *i* after superimposition on the reference frame, and 〈〉 represents the average square distance traveled over residues.

#### PCA analysis

PCA was performed to evaluate domain dynamics within the protein–ligand complex over a 250 ns simulation time scale. Through normal MD simulation mode, it calculates the atomic backbone of the protein–ligand complexes system. The findings were shown following eigen fractions that represent the proportion of variance, collected from a covariance matrix consisting of 20 eigen models.^38^ The first principal component (PC) captured the largest variance within the data, while the second PC encapsulated the second-largest variance orthogonal to the first one. The PCA computation was executed using the R-version 4.4.0 and Bio3D package.

### 2.11. Density functional theory (DFT) calculation

DFT utilizes the principles of quantum mechanics and offers a significantly reliable depiction of electron distribution within a molecule.^39^ This precision allows for the calculation of a variety of molecular properties, including, geometries, energies, and electronic features. The Gaussian 09 W software package was employed to determine several quantum mechanical properties.^40^ The electronic characteristics of the selected molecules were calculated in their singlet ground state, excluding any charge and solvent, employing the Becke-3-parameter Lee–Yang–Parr (B3LYP) method within DFT, using a 6-311g(d,p) basis set for correlation functions.^41^ The DFT approach was used to evaluate the molecule's reactivity by examining various reactivity descriptors, including ionization potential, electron affinity, electronegativity (*χ*), electronic potential (*μ*), chemical hardness (*η*), electrophilicity (*ω*), and chemical softness (*ζ*).^42^

## 3. Results

### 3.1. Re-screening through ML

LightGBM, a machine learning algorithm optimized for gradient boosting, excels at efficiently handling large-scale datasets with high performance. This algorithm assesses compounds based on their inhibitory effects on a specific protein target. Compounds demonstrating inhibition against AChE were utilized to develop several regression models using IC_50_ data. The performance of these models was assessed using *R*^2^ and MSE metrics. After optimizing the model through RFE for feature selection and hyperparameter tuning *via* Grid Search with 5-fold cross-validation, the model achieved an *R*^2^ of 0.87 and an MSE of 0.21 on the test set (Fig. S1†). A high *R*^2^ value indicates a well-fitted model with good performance on test data, while a small MSE value signifies greater accuracy. Among the 80 canonical smiles of compounds, a significant portion (32 compounds) had pIC_50_ values greater than 5.1, as presented in ESI Table 2.†

### 3.2. Molecular docking study

Molecular docking was conducted to determine the binding energies of the phytochemicals to the target protein in structure-based drug discovery and structural biology.^43^ In our intensive research, four compounds, including the control drug, demonstrated higher negative binding energy (greater than −11.26 kcal mol^−1^), representing the top three of the set of 32 compounds, as shown in Table 1. In molecular docking analysis, CID 102267534, CID 15161648, CID 6537302, and CID 12441 showed the highest binding affinity of −11.26, −12.65, and −11.44 kcal mol^−1^, respectively, whereas CID 3152 (donepezil) showed binding energy of −10.76 kcal mol^−1^, which is lower than lead compounds. In addition, all 32 compound's docking scores are presented in ESI Table 3.†

**Table 1:** Molecular docking scores of the identified three compounds along with the control compound

S. no.	PubChem ID	Phytochemical name with 2D structure	Binding affinity (kcal mol^−1^)
1	CID 3152	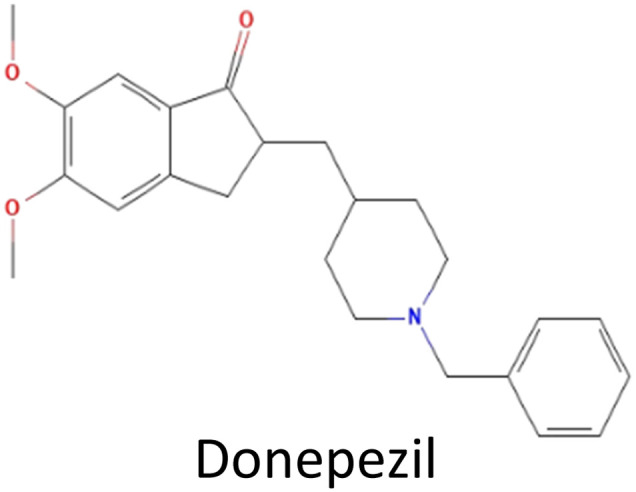	−10.76
2	CID 102267534	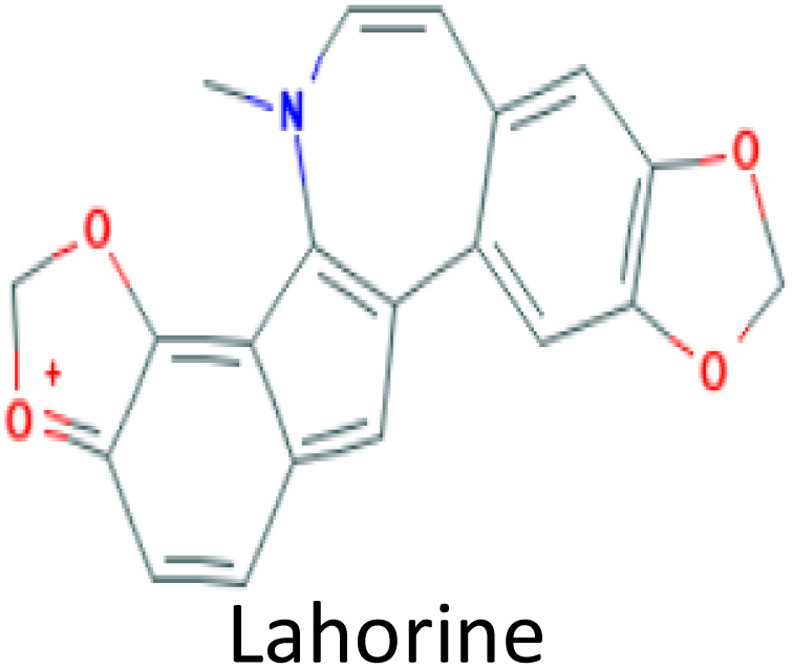	−11.26
3	CID 15161648	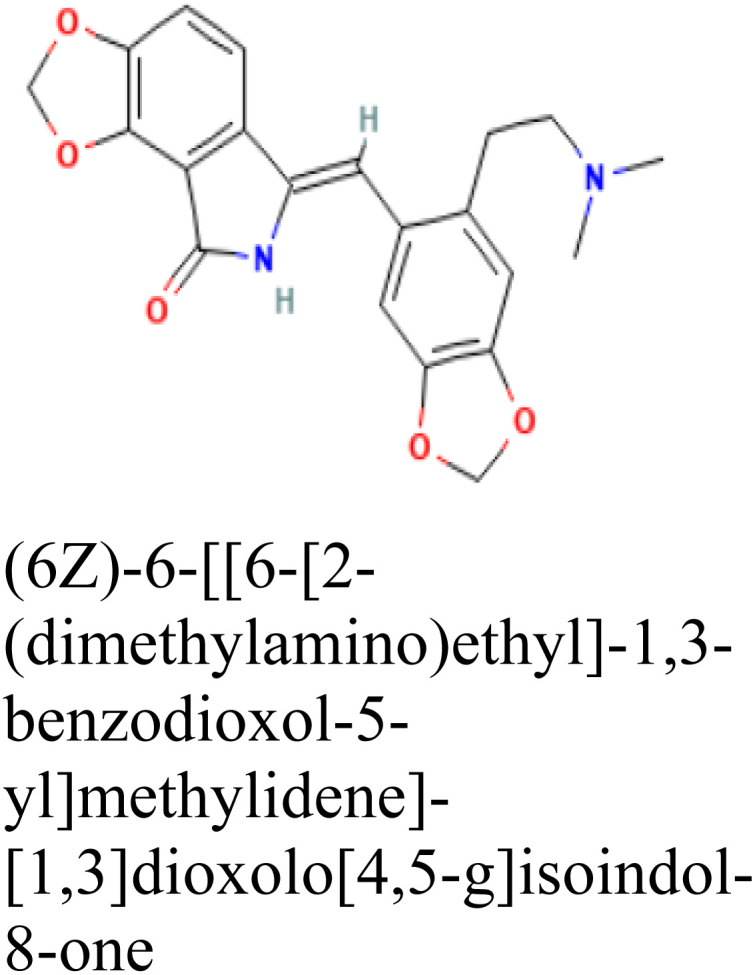	−12.65
4	CID 12441	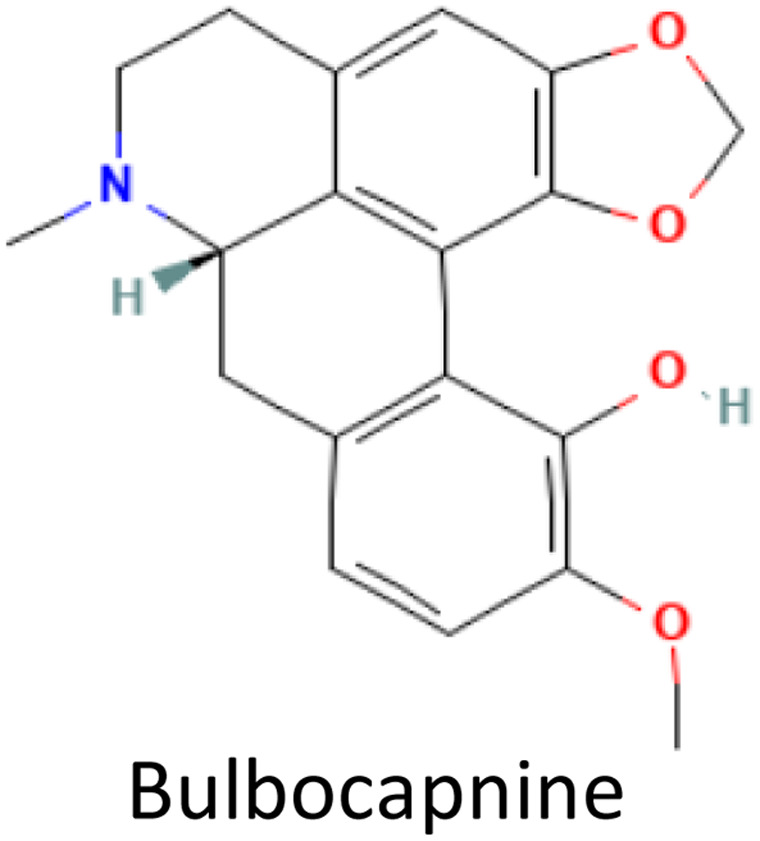	−11.44

### 3.3. Interpretation of protein–ligand interactions

**Fig. 2:**
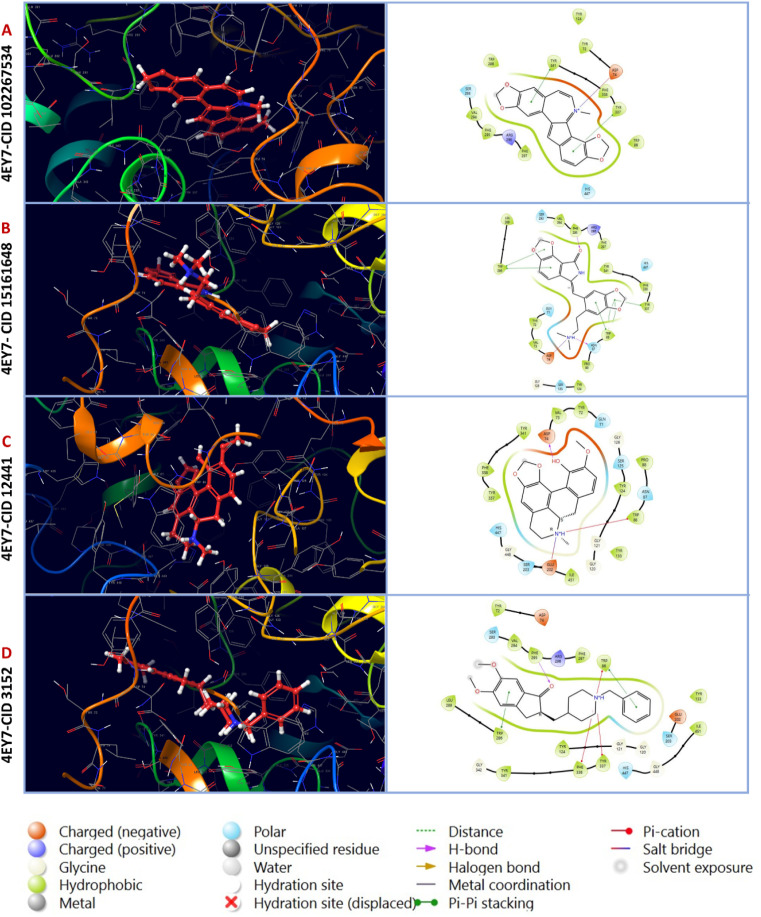
Molecular interactions of interactive ligands with the target receptor (4EY7). The interaction protein–ligand complex in 3D is represented on the left side, whereas the 2D is illustrated on the right side. (A) 4EY7-CID 102267534, (B) 4EY7-CID 15161648, (C) 4EY7-CID 12441, (D) 4EY7-CID 3152.

In this analysis, the interaction between phytochemicals and the targeted receptor was observed using the Schrodinger Suite. Table 2 and Fig. 2 presents the number of hydrogen bonds, hydrophilic interactions, and other types of bonds formed between the amino acid residues of the target protein 4EY7 and the compounds CID 102267534, CID 15161648, CID 6537302, CID 12441, as well as the control compound CID 3152.

**Table 2:** Efficient interactions between amino acid residues of the target protein and the chosen ligands

Protein	Phytochemical (PubChem ID)	H-bonds	Hydrophilic bonds	Others bonds
4EY7	CID 3152	SER239, SER203, HIS447	PHE295	TYR72, ASP74, VAL294, PHE295, ARG296, PHE297, TRP86, LEU289, TRP286, TYR124, GLY121, GLY120, GLU202, TYR133, GLY342, TYR341, PHE338, TYR337, GLY448, ILE451
	CID 102267534	—	SER293, HIS447	VAL294, PHE295, PHE297, TRP286, TYR341, PHE338, TYR337, TRP86, TYR124, TYR72, ASP74, ARG296
	CID 15161648	PHE295, ASN87	SER293, HIS447, GLN71, ASN87, SER125	LEU289, TRP286, TYR72, VAL73, ASP74, GLY 126, TYR124, VAL294, PHE295, ARG296, PHE297, TRY341, PHE338, TYR337, TRP86, PRO88
	CID 12441	ASP74	HIS447, SER203, GLN71, SER125, ASN87	TYR341, TYR337, PHE338, GLY448, GLU202, ILE451, VAL73, TYR72, GLY126, TYR124, GLY121, GLY12O, PRO88, TRP86

### 3.4. MM-GBSA analysis

**Fig. 3:**
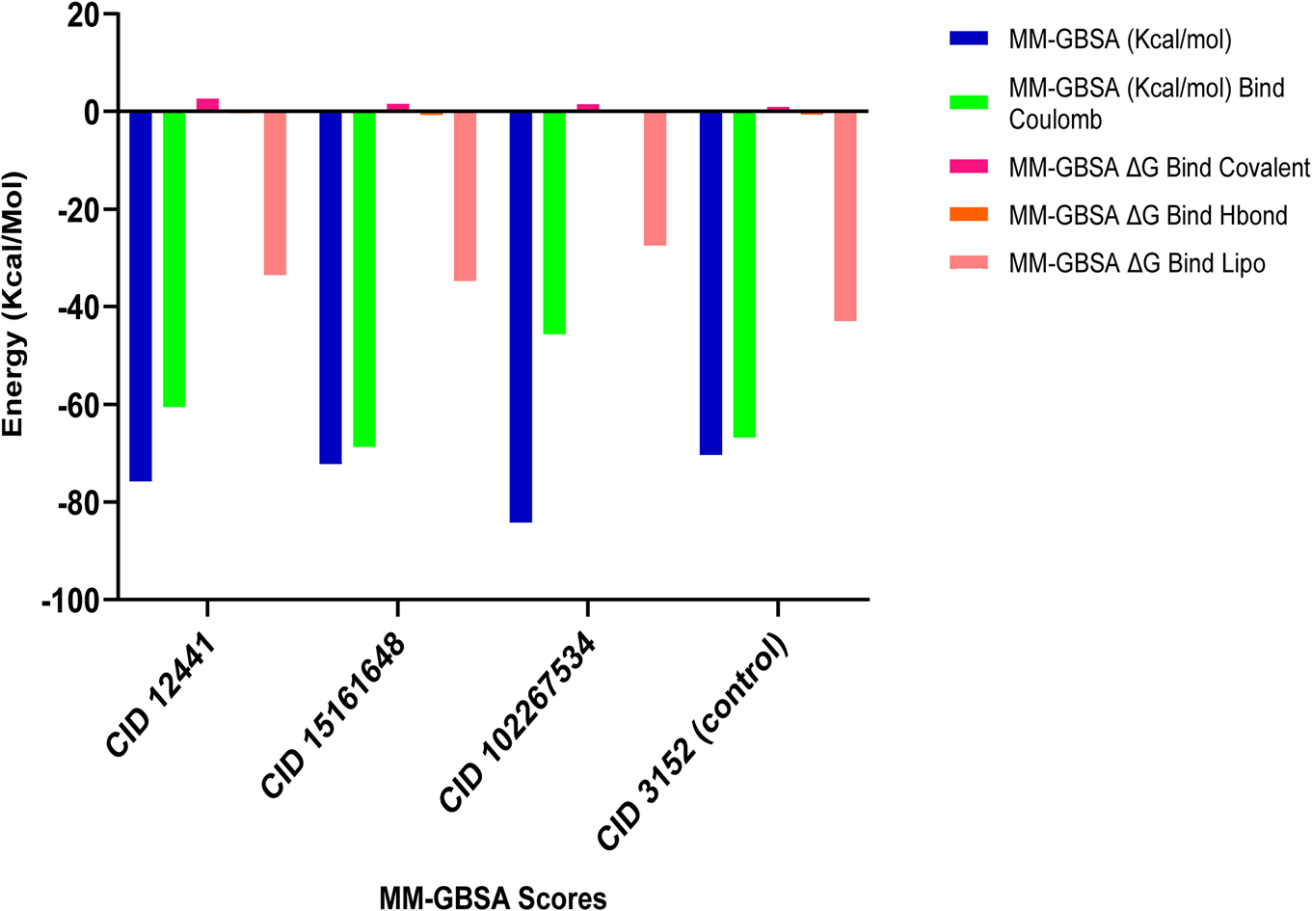
MM-GBSA analysis of CID 12441, CID 15161648, CID 102267534, and CID 3152 (control) with the targeted receptor 4EY7.

MM-GBSA assesses the binding free energy between a drug and its target protein, providing molecular insights into the stability and affinity of the protein–ligand complex.^44^ Our results reveal significant binding affinity, with Δ*G* values indicating strong interactions between the candidate ligands and the target protein, presented in Fig. 3. MM-GBSA analysis of the top three phytochemicals, CID 12441, CID 15161648, and CID 102267534, showed higher binding free energies with the 4EY7 protein, measuring −75.80, −72.27, and −84.29 kcal mol^−1^, respectively, compared to the control compound's binding energy of −80.36 kcal mol^−1^.

### 3.5. Pharmacokinetics and toxicity analysis

Pharmacokinetics is the study of chemicals' flexible movements during their passage through the body. It represents the kinetics of ADME (absorption, distribution, metabolism, and excretion).^45^ The selected phytochemicals showed promising ADME profiling, which is crucial for small molecules to be considered as drugs, presented in Table 3. In addition, the three selected compounds exhibited a lower toxicity profile, which indicates they are safe for administration. An analysis of the toxicity profiles of selected three compounds and control are shown in Table 3.

**Table 3:** Pharmacokinetics and toxicity profile of identified phytochemicals along with control compound

Properties		CID 3152	CID 102267534	CID 15161648	CID 12441
Physicochemical properties	MW (g mol^−1^)	379.49	332.33	380.39	325.36
	Num. heavy atoms	28	25	28	24
	Num. arom. heavy atoms	12	18	12	12
	Num. rotatable bonds	6	0	4	1
	Num. H-bond acceptors	4	4	6	5
	Num. H-bond donors	0	0	1	1
	TPSA	38.77 Å^2^	40.80 Å^2^	69.26 Å^2^	51.16 Å^2^
Lipophilicity	Log *P*_o/w_ (*M* log *P*)	3.06	2.48	1.92	2.16
Water solubility	Log *S* (ESOL)	−4.81	−5.04	−4.03	−4.00
Pharmacokinetics	GI absorption	High	High	High	High
Drug likeness	Lipinski	Yes	Yes	Yes	Yes
Medicinal chemistry	PAINS alert	0	0	0	0
	Drug likeness	0; violation	0; violation	0; violation	0; violation
	Synthetic accessibility	2.73	2.91	3.44	3.77
Toxicity	Hepatotoxicity	Inactive	Inactive	Inactive	Inactive
	Carcinogenicity	Active	Inactive	Inactive	Inactive
	Mutagenicity	Inactive	Inactive	Inactive	Inactive
	Cytotoxicity	Active	Inactive	Inactive	Inactive

### 3.6. Molecular dynamic simulation

The MD simulation of bimolecular structures in solution reveals the dynamics of these structures over a variety of timescales. By providing thermal averages of molecular properties, it simulates the behavior of molecules over time, approximating experimental ensemble averages.^46^ It calculates bulk fluid properties and free energy changes, crucial for processes like ligand binding.^47^ MD also explores accessible conformations, aiding in tasks like ligand docking.^48^ MD simulation was performed on the best complexes to assess their conformational stability and rigidity over a 250 ns period, aimed at identifying potential potent inhibitors. Various parameters including, RMSD, RMSF, *R*_g_, SASA, (P–L) contact, and PCA were monitored for both the apo protein (AChE) and the most favorable ligand complex.

#### RMSD analysis of protein–ligand complexes

**Fig. 4:**
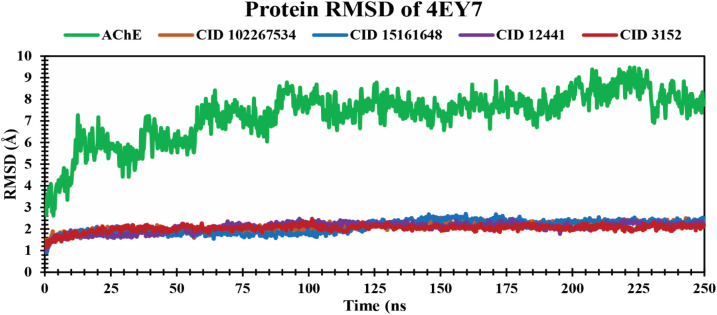
Exhibiting the Cα RMSD values of protein–ligand complexes over a 250 ns simulation period. The compounds CID 102267534, CID 15161648, CID 12441, CID 3152, and apo (AChE) are denoted by orange, blue, violet, red, and green colors, respectively.

The RMSD analysis determines the stability and changes in structural features of the docked protein–ligand complex. Less fluctuation indicates a stable atom backbone and less RMSD is considered favorable evidence for the stability of the docked complex.^49^ The mean value for Cα RMSD of CID 102267534, CID 15161648, and CID 12441 in complex with 4EY7 was 2.11 Å, 2.09 Å, and 2.08 Å, respectively, while apo (AChE) and the control compound CID 3152 showed an average RMSD value of 7.27 Å and 2.03 Å, which is higher, and nearer to lead compounds and represents the structural stability of protein–ligand complexes of the selected compounds throughout the simulation period. The overall RMSD spectrum showed no significant structural shifts, as presented in Fig. 4. This clarifies the protein structure stability and strength of ligand attachment inside the binding site pocket.

#### RMSF analysis of protein–ligand complexes

**Fig. 5:**
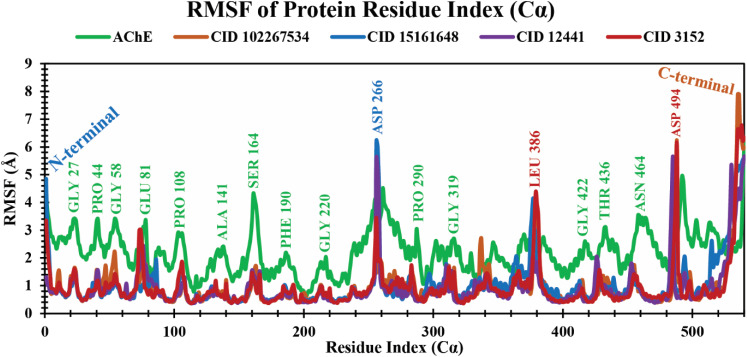
Extracting the RMSF values of 4EY7 in complex with CID 102267534, CID 15161648, CID 12441, and CID 3152 over a 250 ns simulation time frame. The phytochemicals CID 102267534, CID 15161648, CID 12441, CID 3152, and apo (AChE) are denoted by orange, blue, violet, red, and green colors, respectively.

The RMSF is a crucial parameter to estimate the average fluctuations of amino acid residues in the target protein receptor with the selected ligands during the entire simulation time scale. It determines the degree to which atomic positions depart from their mean positions during MD simulations or other dynamic assessments.^50^ The RMSF values for each residue study were calculated over a 250 ns MD simulation time frame. The RMSF analysis showed several increased fluctuating regions, such as GLY27, PRO44, GLY58, GLU81, PRO108, ALA141, SER164, PHE190, GLY220, ASP266, PRO290, GLY319, LEU386, GLY422, THR436, ASN464, and ASP494 amino acid residues at 23, 41, 55, 78, 106, 138, 161, 188, 217, 256, 287, 316, 379, 419, 433, 461, and 488 positions, which are mainly involved in binding of ligands, as shown in Fig. 5. The average RMSF values of 4EY7 in complex with CID 102267534, CID 15161648, and CID 12441 were 0.96 Å, 1.02 Å, and 0.91 Å, respectively, whereas apo (AChE) and the control compound (CID 3152) showed 2.24 Å and 0.94 Å average values, which are higher, and nearer to lead compounds that represent no significant fluctuation in the receptor when formed in complex with the selected lead compounds.

#### *R*_g_ analysis of protein–ligand complexes

**Fig. 6:**
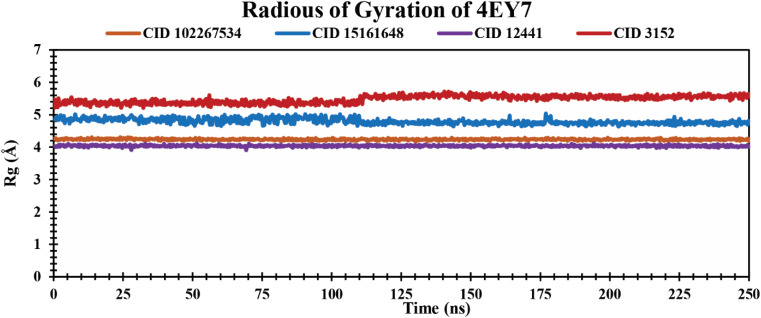
Showing the *R*_g_ values extracted from protein–ligand complexes over a 250 ns simulation period. The phytochemicals CID 102267534, CID 15161648, CID 12441, and CID 3152 in complex with protein are presented by orange, blue, violet, and red colors, respectively.

The *R*_g_ indicates compactness and how atoms are distributed around the axis in protein–ligand complexes over a defined simulation time scale.^51^ It also reinforces the overall shape through a change in compactness or expansion of the system. The lower *R*_g_ values represent a higher degree of compactness.^52^ The *R*_g_ values of protein–ligand complexes over a 250 ns simulation period are graphically demonstrated in Fig. 6. The phytochemicals CID 102267534, CID 15161648, and CID 12441 showed average *R*_g_ values of 4.23 Å, 4.80 Å, and 4.04 Å, respectively. The control CID 3152 showed a 5.47 Å average *R*_g_ value when formed in a complex with 4EY7 protein which is higher than the lead phytocompounds. These results suggest that CID 102267534, CID 15161648, and CID 12441 exhibit no major structural shift in the active site of protein after binding compared to the control compound CID 3152.

#### SASA analysis of protein–ligand complexes

**Fig. 7:**
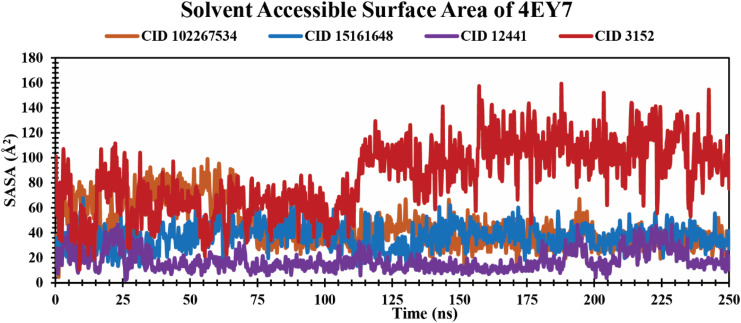
Illustrating the SASA values of protein–ligand complexes over a 250 ns simulation time scale. The phytocompounds CID 102267534, CID 15161648, CID 12441, and CID 3152 in complex with protein are presented by orange, blue, violet, and red colors, respectively.

SASA is the measurement of protein surface area easily accessible to solvent molecules.^53^ It represents molecular insights into conformational fluctuations and the interaction of ligands with protein macromolecules which are illustrated in Fig. 7. In this study, all the lead phytochemicals CID 102267534 CID 15161648 and CID 12441 in complex with the target receptor (4EY7) demonstrated a relatively consistent SASA trajectory characterized by minor fluctuations over the 250 ns simulation period. In contrast, CID 3152 had the largest SASA throughout the simulation, indicating the maximum surface area exposed to solvent.

#### P–L contact analysis

**Fig. 8:**
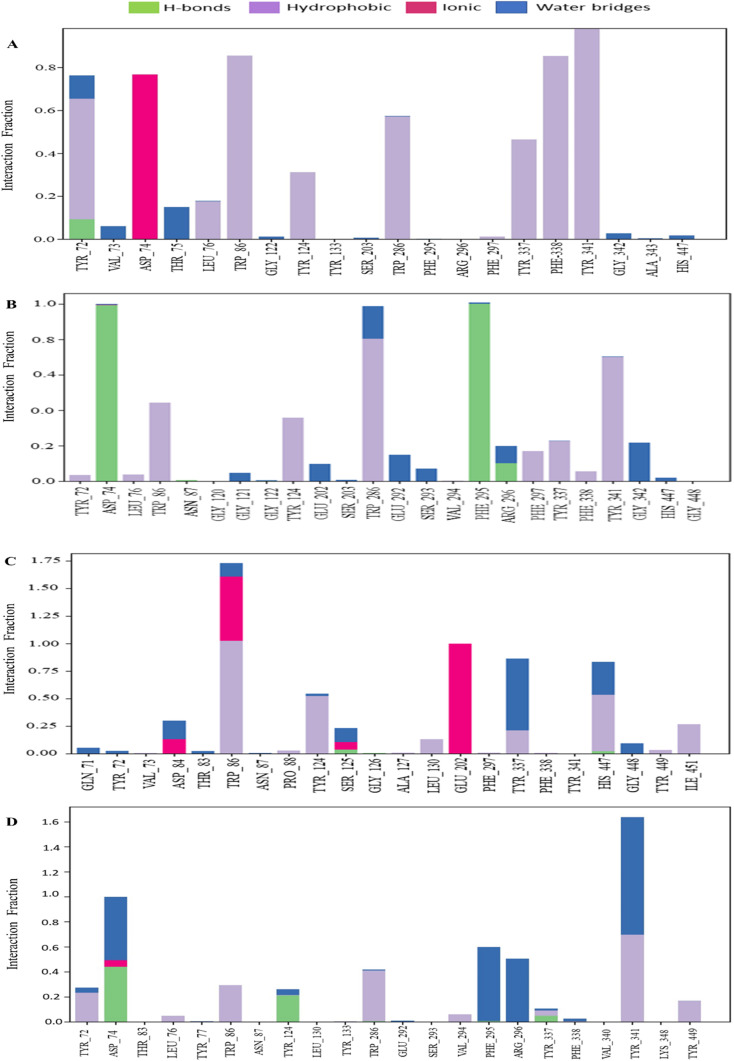
The bar graph depicts the interactions between phytochemicals and proteins observed over a 250 ns simulation. The interactions of CID 102267534, CID 15161648, CID 12441, and CID 3152 with the receptor (4EY7) are denoted by (A)–(D), respectively.

The examination of protein–ligand complex structures and their intermolecular interactions was carried out using the Simulation Interactions Diagram (SID).^54^ The various parameters, including hydrogen bonding, ionic bonding, noncovalent interactions (hydrophobic bonding), and water bridge bonding of the selected phytochemicals CID 12441, CID 15161648, and CID 102267534, along with the control CID 3152 with the receptor (4EY7), were considered in the study, and the findings are shown in Fig. 8. The CID 12441 depicted significant interactions with the amino acid residues of 4EY7 protein during the 250 ns simulation, including H-bonds at SER125 residue, hydrophobic bonds at TRP86, PRO88, TYR124, TRP286, LEU130, TYR337, HIS447, TYR449, and ILE451 residues, ionic bonds at ASP84, TRP86, SER125, GLU202 residues, and water bridge bonds at GLN71, TYR72, THR83, ASP84, TRP86, TYR124, SER125, TYR337, HIS447, and GLU448 residues.

#### Principle component analysis (PCA)

**Fig. 9:**
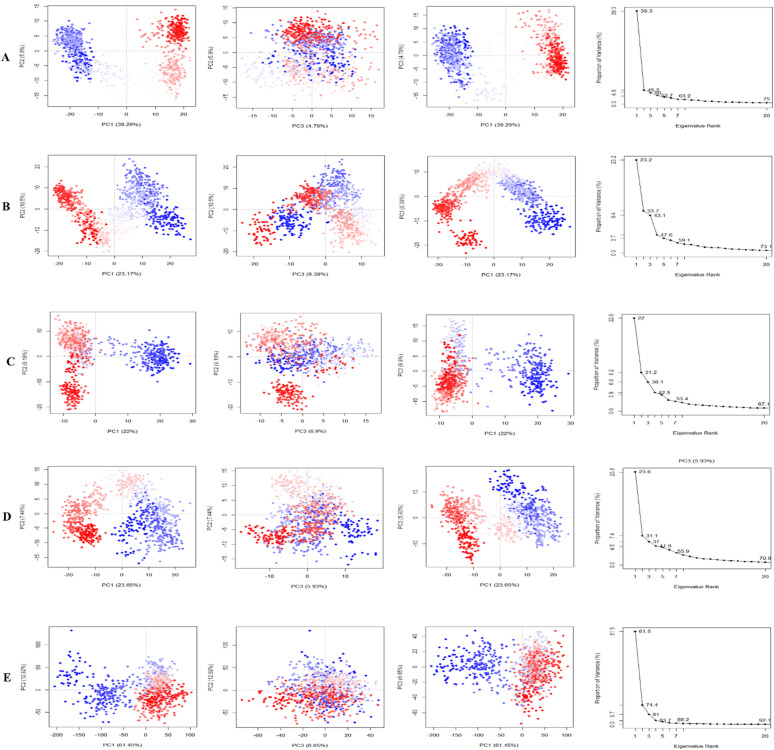
Illustrating the principal component analysis (PCA) results of apo and protein–ligand complexes over a 250 ns simulation period. The PCA of CID 15161648, CID 102267534, CID 12441, and CID 3152 with the receptor complexes, and apo (4EY7), are denoted by (A)–(E), respectively.

PCA was employed to investigate the domain dynamics of the protein–ligand complexes over a 250 ns simulation time scale. The results, expressed as eigen fractions, represent the variance proportion calculated from a covariance matrix containing 20 eigen models. PCA calculates the atomic backbone of the complex system using three conformations, PC1, PC2, and PC3, through normal MD mode. In particular, the PCA showed conformational shifts across all clusters with the red zone indicating the least fluctuating movements, while the white region depicted intermediate movements, and the blue region showed the most significant movements. The conformational transformations of the apo (4EY7), 4EY7-CID 3152 (control), 4EY7-CID 15161648, 4EY7-CID 12441, and 4EY7-CID 102267534 systems were represented in Fig. 9. PCA scatter plots were generated by projecting the simulated trajectories onto the two-dimensional subspace denoted by the first three eigenvectors (PC1, PC2, and PC3), as shown in Fig. 9. The continuous color spectrum represents the progression of time, from blue (starting timescale) to white (intermediate timescale) to red (ultimate timescale). The RMSF of residue contribution to PCA is depicted in Fig. 9 (left and right sides), with the black and blue lines representing PC1 and PC2, respectively.

### 3.7. Density functional theory (DFT) calculation

**Fig. 10:**
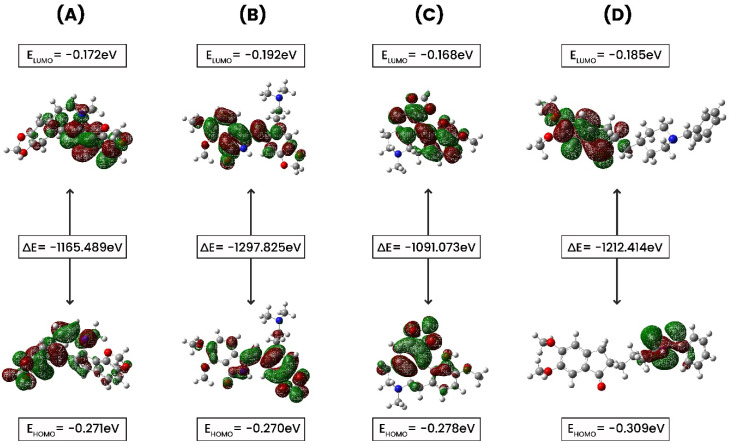
The ground state molecular orbital distribution plots of (A) CID 102267534, (B) CID 15161648, (C) CID 12441, and (D) CID 3152.

FMO (Frontier Molecular Orbital) studies is a computational method in quantum chemistry to calculate and analyze the energies, electron distributions, and shapes of the highest occupied molecular orbital (HOMO) and the lowest unoccupied molecular orbital (LUMO) in a molecule.^55^ A FMO calculation evaluates the electron affinity, and ionization potential for compounds CID 102267534, CID 15161648, CID 12441, and CID 3152. In this study, electronic descriptor evaluation encompasses *E*_HOMO_, *E*_LUMO_, Δ*E*_gap_, electron affinity, ionization potential, electronegativity, hardness, softness, chemical potential, electrophilicity, and electronic potential, as delineated in Fig. 10 and Table 4. The differences in Eh indicate variations in bonding interactions, and stability among these compounds, with higher energy values suggesting greater reactivity. CID 12441 had the highest energy value at −1091.073 Eh, while CID 15161648 had the lowest at −1297.825 Eh. Conversely, CID 3152 exhibited the highest dipole moment at 3.876 D, whereas CID 102267534 had the lowest at 1.0599 D. CID 12441 and CID 15161648 had dipole moments of 3.0647 D and 2.5937 D, respectively. Variations in dipole moment values indicate differences in polarity and charge distribution. CID 3152 showed higher polarity whereas CID 102267534 had less polarity.

**Table 4:** The data of chemical reactivity descriptors of our selected four phytochemicals were calculated employing the DFT B3LYP/3-21G* basis set method

Features	CID 102267534	CID 15161648	CID 12441	CID 3152
Electronic energy (Eh)	−1165.489	−1297.825	−1091.073	−1212.414
Dipole moment (D)	1.059	2.593	3.064	3.876
*E*_HOMO_ (eV)	−0.271	−0.270	−0.278	−0.309
*E*_LUMO_ (eV)	−0.172	−0.192	−0.168	−0.185
Δ*E*_gap_ (eV)	0.099	0.077	0.110	0.124
Ionization potential (eV)	0.271	0.270	0.278	0.309
Electron affinity (eV)	0.172	0.192	0.168	0.185
Electronegativity (eV)	−0.221	−0.231	−0.223	−0.247
Chemical potential (eV)	0.221	0.231	0.223	0.247
Hardness (eV)	−0.049	−0.038	−0.055	−0.062
Softness (eV^−1^)	−20.106	−25.816	−18.129	−16.109
Electronic potential (eV)	−0.221	−0.231	−0.223	−0.247
Electrophilicity (eV)	−0.494	−0.692	−0.451	−0.493

The *E*_HOMO_ values ranged from −0.27027 to −0.3095 eV, with CID 3152 having the lowest and CID 15161648 the greatest. *E*_LUMO_ values ranged from −0.16813 to −0.1928 eV, with CID 15161648 having the lowest and CID 12441 the highest. The Δ*E*_gap_ varied from 0.07747 to 0.12415 eV, with CID 3152 exhibiting the widest energy gap. Ionization potential values spanned from 0.27027 eV to 0.3095 eV. Electronegativity values varied between −0.221845 and −0.247455 eV, with CID 102267534 having the highest value. Chemical potential values were between 0.221845 and 0.247455 eV. Hardness values ranged from −0.03874 to −0.062075 eV, with CID 15161648 being the hardest. Softness values ranged from −16.1095 to −20.1066 eV^−1^, and electronic potential values varied between 0.221845 and 0.247455 eV. Electrophilicity values ranged from −0.4519 to −0.6920 eV.

The identified compounds displayed different electronic structures than the control. Specifically, CID 12441 had the highest energy value and *E*_LUMO_ values, CID 3152 had the highest dipole moment and the widest energy gap, CID 15161648 had the highest *E*_HOMO_ values, and CID 102267534 had the highest electronegativity. Additionally, these selected compounds differed from the control compound in terms of electrophilicity, and electronegativity, representing differences in their potential interactions, and chemical reactivity with the reference molecule, CID 3152.

## 4. Discussion

AD is a progressive brain disorder characterized by impairment in critical thinking and behavior and memory loss that hampers normal living.^56^ ACh was broken into acetyl and choline by AChE occurs during neural transmission on the post-synaptic membrane. This breakdown of ACh disrupts normal neuronal transmission in the synaptic cleft, resulting in the development of cholinergic AD symptoms.^57^ The development of new therapeutics for treating AD can be achieved by blocking AChE activity. Blockers of AChE inhibit ACh breakdown and, in turn, enhance neurotransmitter function.^58^ Natural products are well-known as effective sources of medications for a variety of human diseases and have been used since ancient times.^59–62^ In this study, we utilized 2500 compounds from 25 traditionally used medicinal plants.

Virtual screening and computer-aided drug design (CADD) have emerged as critical tools for identifying and discovering novel therapeutic chemicals since they hold many advanced computational techniques.^63^ Employing advanced techniques such as virtual screening through ML, molecular docking, MM-GBSA, and MD simulation not only reduces experiment duration and costs but also enhances prediction accuracy, making them indispensable in CADD, particularly for virtual screening of natural bioactive phytochemicals library to identify potential drug candidates.^64^ In this study, filtering unwanted substructures through physicochemical analysis reduced 2500 compounds to 80, and subsequent re-screening using machine learning (ML) identified 32 compounds with promising IC_50_ values.

Molecular docking is an essential tool in drug discovery and development, facilitating the prediction of binding affinities between small molecules and the target macromolecules. The molecular docking study unveiled three compounds, CID 102267534, CID 15161648, and CID 12441, exhibiting binding energies exceeding −11.26 kcal mol^−1^, suggesting a stronger interaction with AChE. These findings are in line with the concept that lower binding energies generally correspond to more stable and potent interactions.^65^ One notable aspect of the binding interactions observed in this study is the role of hydrogen, hydrophobic, and other interactions (Fig. 2). These interactions likely contribute to the structural stability of the protein–ligand complexes and their potential inhibitory effects on AChE. In the MM-GBSA analysis, the lowest Δ*G* bind score (the most negative score) represents the best Δ*G* bind score.^66^ Among the docked compounds for AChE, the three lead compounds, CID 102267534, CID 15161648, and CID 12441, depicted superior Δ*G*-bind scores compared to the control compounds, highlighting their efficacy as illustrated in Fig. 3. The ADME/T analysis revealed that all three ligands adhered to Lipinski's rules, which are essential for identifying potential lead compounds. In pharmacokinetics and toxicity analysis, the selected three lead compounds CID 102267534, CID 15161648, and CID 12441 showed satisfactory results, as shown in Table 3.

An MD simulation is applied to determine the stability of a protein when complexed with its ligand. Additionally, it determines the structural stability and rigidity of protein–ligand complexes over a specific period in an artificial environment, such as the human body.^67^ The MD simulations performed in this study provided valuable insights into the conformational stability and dynamics of the AChE protein–ligand complexes over a 250 ns timescale. The analysis of key features, including RMSD, RMSF, *R*_g_, SASA, protein–ligand (P–L) contact, and PCA, shed light on the structural characteristics and behavior of these complexes. RMSD calculations reflect protein–ligand complex stability, whereas RMSF values reveal residual fluctuations during ligand binding.^67^ Over a 250 simulation period, the three identified compounds, CID 102267534, CID 15161648, and CID 12441, showcased lower RMSD, and RMSF values when complexed with the receptor, representing the structural stability of the complexes, presented in Fig. 4 and 5. The lower *R*_g_ value suggests a high degree of compactness, whereas the higher value indicates that the chemicals dissociate from the protein.^68^ Compared to the CID 3152–protein complex, the complexes formed by the lead three compounds exhibited superior *R*_g_ values, represented in Fig. 6. Lower SASA values show tightly packed complexes of amino acids and water molecules, while higher values imply less stable structures. In this instance, the complexes formed by CID 102267534, CID 15161648, and CID 12441 with the receptor displayed lower SASA values in comparison to the complexes formed by the protein with CID 3152, illustrated in Fig. 7. Using SID, the arrangement of amino acid residues in the protein when bound to the chosen ligand was examined, alongside their molecular interactions. Additionally, the selected phytochemicals CID 102267534, CID 15161648, and CID 12441 established stronger hydrophobic bonds, increased hydrogen bonds, and water bridge bonds with AChE compared to the control ligand CID 3152, represented in Fig. 8.

The low level of variability depicted by PC3 (6.65%) for apo (4EY7), compared to PC1 (61.45%) and PC2 (12.92%), demonstrates that the apo (4EY7) is highly stable and the structure is compact. In addition, RMSF analysis on the PCA showed that the flexibility of PC1 and PC2 decreased compared to 4EY7-CID 3152 (control). For 4EY7-CID 15161648, the low variability of PC3 (4.79%) compared to PC1 (39.29%) and PC2 (5.9%) also indicates compact binding. Similarly, 4EY7-CID 12441 showed low variability in PC3 (6.9%) relative to PC1 (22%) and PC2 (9.18%), indicating compact binding. Lastly, for 4EY7-CID 102267534, the low variability of PC3 (9.39%) compared to PC1 (23.17%) and PC2 (10.5%) indicates compact binding, presented in Fig. 9.

FMOs were utilized to evaluate the kinetics and identify locations where proteins might fold into active pharmacophores.^69^ Using DFT, we determined the orbital geometry of our identified compounds. HOMO refers to an orbital with electron-dense regions, whereas LUMO refers to an orbital with electron-deficient regions. HOMO and LUMO are used to define the electron-donating and electron-accepting characteristics of chemicals. Another important parameter to consider is the energy gap, which denotes the difference between the highest occupied molecular orbital (HOMO) and the lowest unoccupied molecular orbital (LUMO) energies. This disparity indicates kinetic stability and intramolecular charge transfer.^70^ Compounds with a large energy gap show increased kinetic stability, and decreased chemical reactivity, while those with a shorter energy gap show lowered kinetic stability and higher reactivity. In this analysis, the HOMO and LUMO energies of the selected compounds along with control (CID 3152) were calculated using the quantum mechanical DFT method while CID 102267534 and CID 12441 showed a large energy gap compared to CID 3152 (control), indicating high kinetic stability, as shown in Fig. 10.

Considering the thorough analysis conducted, CID 102267534, CID 15161648, and CID 12441 emerge as promising candidates for addressing AD. Nevertheless, extensive laboratory trials are imperative to ascertain the anti-AChE properties of these phytochemicals, offering potential alternatives in AD treatment.

## 5. Conclusion

This study isolated 81 phytochemicals from 25 medicinal plants using machine learning, focusing on their potential as anti-AD drugs targeting acetylcholinesterase (AChE). Among them, three compounds (CID 102267534, CID 15161648, and CID 12441) were selected based on molecular docking results, followed by ADME and toxicity analysis confirming their pharmacokinetics and safety. MD simulation validated the structural stability of these compounds at AChE's active site, suggesting their potential as inhibitors. However, further *in vivo* and *in vitro* studies are required to validate their efficacy against AChE.

## Abbreviations

AD: Alzheimer's disease
AChE: Acetylcholinesterase enzyme
ML: Machine learning
IMPPAT: Indian Medicinal Plants, Phytochemistry, and Therapeutics
MM-GBSA: Molecular mechanics-generalized born surface area
ADME: Absorption, distribution, metabolism, and excretion
SID: Simulation interaction diagram
RMSD: Root mean square deviation
RMSF: Root mean square fluctuation
*R*_g_
: Radius of gyration
SASA: Solvent accessible surface area

## Data availability

Data will be made available on request.

## Author contributions

Md. Tarikul Islam: project design, data generation, software, formal analysis, writing, and reviewing manuscript. Md. Aktaruzzaman: data generation, software, supervision, formal analysis, writing. Ahmed Saif: data analysis, formal analysis, and writing. Al Riyad Hasan: visualization, and writing. Md. Mehedi Hasan Sourov: visualization, and data curation. Bratati Siktar: visualization, and writing. Saira Rehman: critically reviewed the manuscript, writing and review. Afrida Tabassum: writing. Syed Abeed-Ul-Haque: data curation, writing. Mehedi Hasan Sakib: resources, data curation. Md. Muntasir Alam Muhib: data curation. Md. Ali Ahasan Setu: writing and review. Faria Tasnim: writing. Rifat Rayhan: data curation. Mohamed M. Abdel-Daim: writing and review. Md. Obayed Raihan, PhD: validation, editing manuscript, supervision.

## Conflicts of interest

The authors have disclosed no conflicts of interest.

## Supplementary Material

RA-014-D4RA05073H-s001

RA-014-D4RA05073H-s002

RA-014-D4RA05073H-s003

RA-014-D4RA05073H-s004

RA-014-D4RA05073H-s005

RA-014-D4RA05073H-s006
