# Supplementary material for: Identification of acetylcholinesterase inhibitors from traditional medicinal plants for Alzheimer's disease using in silico and machine learning approaches
Source: RSC Adv. 2024 Oct 31;14(47):34620–36. doi: 10.1039/d4ra05073h (PMC11526779; doi:10.1039/d4ra05073h)
Supplement: RA-014-D4RA05073H-s001 [file RA-014-D4RA05073H-s001.pdf]

Supplementary Information (SI) for RSC Advances.
This journal is © The Royal Society of Chemistry 2024

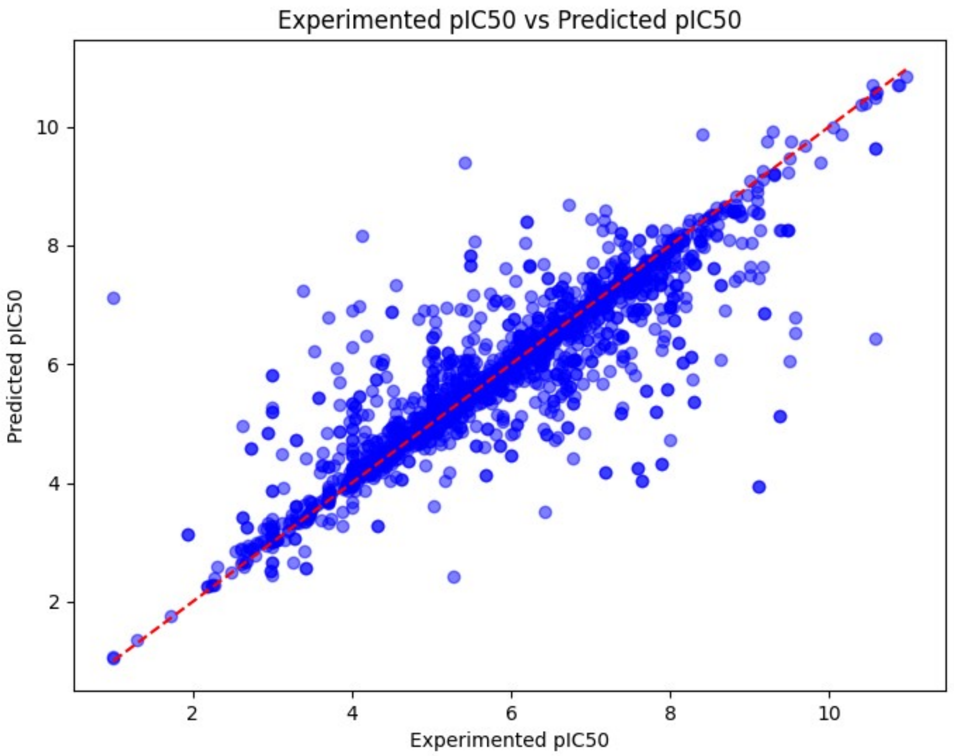

Fig. S1 Plot comparing the experimental pIC50 values to the predicted pIC50 values obtained from the model using fingerprint descriptors.
