# Supplementary material for: Identification of acetylcholinesterase inhibitors from traditional medicinal plants for Alzheimer's disease using in silico and machine learning approaches
Source: RSC Adv. 2024 Oct 31;14(47):34620–36. doi: 10.1039/d4ra05073h (PMC11526779; doi:10.1039/d4ra05073h)
Supplement: RA-014-D4RA05073H-s003 [file RA-014-D4RA05073H-s003.pdf]

Supplementary Information (SI) for RSC Advances.
This journal is © The Royal Society of Chemistry 2024

S.I.	Local Name	Scientific name	Traditional Use	Citations
1.	Khudijam	*Cleistocalyx nervosum*	Antidiabetic, anti-neuroinflammatory, and activity	[41]
2.	Kwao Krua	*Pueraria mirifica*	Anti-mutagenic, and antioesteroporosis activity	[42], [43]
3.	Snake jasmine	*Rhinacanthus nasutus*	Anti-inflammatory, and anti-obesity activity	[44], [45]
4.	Sheora	*Streblus asper*	Anticancer, anti-allergic, antifilarial, anti-parkinson, hepto-protective, antineoplastic, anti-allergic, and anti-inflammatory activity	[46],
5.	Brahmi	*Bacopa monnier*	Anti-convulsant, anti-depressant, analgesic, hepatoprotective, anti-inflammatory, anxiolytic, anti-microbial, anti-ulcerogenic, anti-h. Pylori, apoptogenic, anti-neoplastic, and immunostimulatory activity	[47]
6.	Haritaki	*Terminalia chebula*	Antipyretic, astringent, spasmolytic, expectorant, antiviral, antiasthmatic, and hypoglycemic activity	[47]
7.	Satamuli	*Asparagus racemosus*	Anticancer, and anti-inflammation activity	[48]
8.	Bitter fennel, sweet fennel	*Foeniculum vulgare*	Anti-ulcer, antinsomnia, and anti-cancer activity	[49]
9.	Neem	*Azadirachta indica* Juss.	Insecticide, anti-diabetic, and anti-cancer activity	[50], [51]
10.	Picrorhiza, katuka, kutki	*Picrorhiza kurroa*	Antipyretic, anti-arthritic, and antidiarrheic activity	[52]
11.	Bonshapla, Bon mosur	*Fumaria indica*	Antiviral, antipyretic, and anthelmintic activity	[53]
12.	Bharbharra Jawasa	*Alhagi pseudalhagi*	Antibacterial, anti-urolithic, anti-ulcer, anti-anginal, antipyretic, antioxidant, diuretic, inflammation, and anti-arthritic activity	[54]
13.	Rasayana herb	*Pluchea lanceolata*	Anti-inflammatory, anti-arthritic, anticancer, antipyretic, antioxidant, and antimalarial activity.	[55]
14.	Agnimantha	*Premna mucronata*	Antibacterial, anti-inflammatory, and antioxidant activity	[56]

15.	Beula	*Semecarpus anacardium*	Anti-artherosclerotic, anti-inflammatory, antioxidant, neuroprotective, hypoglycemic, and anti-carcinogenic activity	[57]
16.	Lal berela	*Sida cordifolia*	Anti-inflammatory and analgesic, anticancer, diuretic, hypoglycemic, and anthelmintic activity	[58]
17.	Gulancha	*Tinospora cordifolia*	Anti-diabetic, anti-cancer, immunomodulatory, antipyretic, hypolipidaemic, anti-arthritic, and anti-viral activity	[59]
18.	Hog plum	*Spondias mombin*	Antioxidant, anti-inflammatory, gastroprotective, antitumor and antiangiogenic, and antiulcer activity.	[60]
19.	Guinea pepper	*Aframomum melegueta*	Anti-diabetic, anti-tumor, anti-cancer, and anti-inflammatory activity	[61]
20.	Poison devil's-pepper	*Rauwolfia vormitoria*	Antipsychotic effect, anti-inflammatory, antipyretic, anti-cancer, and anti-diabetic activity	[62]
21.	African oil palm	*Elaeis guineensis*	Anti-inflammatory, anti-bacterial, anti-cancer, anti-oxidant activity, and anti-diabetic activity	[63]
22.	Bellyache bush physicnut	*Jatropha curcas*	Anti-bacterial, anti-fungal, and analgesic activity	[64]
23.	Ceylon spinach	*Talinum triangulare*	Antioxidant activity	[65]
24.	Flat crown	*Albizia adianthifolia*	Anti-diabetic, analgesic, and antiviral activity	[66]
25.	Coral plant	*Jatropha multifida*	Antimicrobial, anti-inflammatory and antioxidant activity	[67]
