# Supplementary material for: Identification of acetylcholinesterase inhibitors from traditional medicinal plants for Alzheimer's disease using in silico and machine learning approaches
Source: RSC Adv. 2024 Oct 31;14(47):34620–36. doi: 10.1039/d4ra05073h (PMC11526779; doi:10.1039/d4ra05073h)
Supplement: RA-014-D4RA05073H-s004 [file RA-014-D4RA05073H-s004.pdf]

Supplementary Information (SI) for RSC Advances.
This journal is © The Royal Society of Chemistry 2024

SL No.	Title (PubChem ID)	Phytochemicals Name	pIC50
1	27208	3-Cyclohexen-1-ol 1-(15-dimethyl-4-hexenyl)-4-methyl	5.766400
2	3152	Donepezil	5.497164
3	4114	Methoxsalen	5.462993
4	5280863	Kaempferol	5.453437
5	12441	Bulbocapnine	5.325781
6	1550607	Auraptene	5.309136
7	102267534	13-Methyl-571719-tetraoxa-13-azoniahexacyclo[12.10.0.0210.048.01523.01620]tetracosa-1(24)24(8)9111315(23)16(20)21-nonaene	5.308796
8	15161648	(6Z)-6-[[6-[2-(dimethylamino)ethyl]-13-benzodioxol-5-yl]methylidene]-[13]dioxolo[45-g]isoindol-8-one	5.292476
9	1549992	Bisabolol	5.295872
10	11019992	o-Menth-2-ene 4-isopropylidene-1-vinyl	5.295872
11	445070	trans-trans-Farnesol	5.289255
12	5284507	trans-Nerolidol	5.285173
13	12300148	6-Epi-beta-bisabolol	5.284777
14	441005	(+)-delta-Cadinene	5.254739
15	6432404	(+)-gamma-Cadinene	5.254739
16	10248	Elemicin	5.231153
17	3085362	Gigantol	5.223878
18	1549107	cis-cis-Farnesol	5.218536
19	1549108	(2Z,6E)-Farnesol	5.218536
20	1549109	(E,Z)-Farnesol	5.218536
21	24838	Hexyl 2-methylbutanoate	5.204709
22	638014	beta-Ionone	5.207413
23	10856614	alpha-Selinene	5.193275
24	442393	beta-Selinene	5.193275
25	1742210	beta-CARYOPHYLLENE OXIDE	5.188125
26	6432312	(1S,2S)-1-ethenyl-1-methyl-4-propan-2-ylidene-2-prop-1-en-2-ylcyclohexane	188678

27	6537302	(3Z)-3-[[6-[2-(dimethylamino)ethyl]-13-benzodioxol-5-yl]methylidene]-67-dimethoxyisoindol-1-one	5.188678
28	10228	Osthol	5.176082
29	1803558	Meranzin	5.147846
30	5281515	Caryophyllene	5.148371
31	12309449	(RR)-1-isopropyl-4-methyl-3-(prop-1-en-2-yl)-4-vinylcyclohexene	5.146411
32	86374)	Allixin	5.138466
