# Supplementary material for: Identification of acetylcholinesterase inhibitors from traditional medicinal plants for Alzheimer's disease using in silico and machine learning approaches
Source: RSC Adv. 2024 Oct 31;14(47):34620–36. doi: 10.1039/d4ra05073h (PMC11526779; doi:10.1039/d4ra05073h)
Supplement: RA-014-D4RA05073H-s005 [file RA-014-D4RA05073H-s005.pdf]

Supplementary Information (SI) for RSC Advances.
This journal is © The Royal Society of Chemistry 2024

| SN | Title (PubChem id) | Phytochemicals names | Docking scores |
|---|---|---|---|
| 1 | 27208 | 3-Cyclohexen-1-ol, 1-(1,5-dimethyl-4-hexenyl)-4-methyl- | -5.22589 |
| 2 | 4114 | Methoxsalen | -6.81928 |
| 3 | 3152 | Donepezil | -10.76 |
| 4 | 5280863 | Kaempferol | -6.80479 |
| 5 | 1803558 | Meranzin | -6.02545 |
| 6 | 12441 | Bulbocapnine | -11.44 |
| 7 | 3085362 | Gigantol | -6.53888 |
| 8 | 1742210 | beta-caryophyllene oxide | -5.0152 |
| 9 | 10228 | Osthol | -5.79098 |
| 10 | 102267534 | 13-Methyl-5,7,17,19-tetraoxa-13-azoniahexacyclo[12.10.0.02,10.04,8.015,23.016,20]tetracosa-1(24),2,4(8),9,11,13,15(23),16(20),21-nonaene | -11.26 |
| 11 | 10248 | Elemicin | -5.34565 |
| 12 | 445070 | trans,trans-Farnesol | -7.051432 |
| 13 | 1549107 | cis,cis-Farnesol | -2.76703 |
| 14 | 1549108 | (2Z,6E)-Farnesol | -2.43306 |
| 15 | 1549109 | (E,Z)-farnesol | -3.08826 |
| 16 | 24838 | Hexyl 2-methylbutanoate | -1.18347 |
| 17 | 1549992 | Bisabolol | -6.46758 |
| 18 | 11019992 | o-Menth-2-ene, 4-isopropylidene-1-vinyl- | -5.35341 |
| 19 | 15161648 | (6Z)-6-[[6-[2-(dimethylamino)ethyl]-1,3-benzodioxol-5-yl]methylidene]-[1,3]dioxolo[4,5-g]isoindol-8-one | -12.65 |
| 20 | 1550607 | Auraptene | -7.14465 |
| 21 | 441005 | (+)-delta-Cadinene | -5.66826 |
| 22 | 6432404 | (+)-gamma-Cadinene | -5.71785 |
| 23 | 10856614 | alpha-Selinene | -5.04896 |
| 24 | 442393 | beta-Selinene | -5.96309 |
| 25 | 6432312 | (1S,2S)-1-ethenyl-1-methyl-4-propan-2-ylidene-2-prop-1-en-2-ylcyclohexane | -4.68998 |
| 26 | 6537302 | (3Z)-3-[[6-[2-(dimethylamino)ethyl]-1,3-benzodioxol-5-yl]methylidene]-6,7-dimethoxyisoindol-1-one | -8.018543 |
| 27 | 12309449 | (R,R)-1-isopropyl-4-methyl-3-(prop-1-en-2-yl)-4-vinylcyclohexene | -4.67205 |
| 28 | 12300148 | 6-Epi-beta-bisabolol | -5.2258 |
| 29 | 86374 | Allixin | -6.3373 |
| 30 | 638014 | beta-Ionone | -6.52592 |
| 31 | 5284507 | trans-Nerolidol | -2.29071 |
| 32 | 5281515 | Caryophyllene | -5.19744 |
